# JEDI: circular RNA prediction based on junction encoders and deep interaction among splice sites

**DOI:** 10.1093/bioinformatics/btab288

**Published:** 2021-07-12

**Authors:** Jyun-Yu Jiang, Chelsea J -T Ju, Junheng Hao, Muhao Chen, Wei Wang

**Affiliations:** Department of Computer Science, University of California, Los Angeles, CA 90024, USA; Department of Computer Science, University of California, Los Angeles, CA 90024, USA; Department of Computer Science, University of California, Los Angeles, CA 90024, USA; Department of Computer Science, University of Southern California, Los Angeles, CA 90007, USA; Department of Computer Science, University of California, Los Angeles, CA 90024, USA

## Abstract

**Motivation:**

Circular RNA (circRNA) is a novel class of long non-coding RNAs that have been broadly discovered in the eukaryotic transcriptome. The circular structure arises from a non-canonical splicing process, where the donor site backspliced to an upstream acceptor site. These circRNA sequences are conserved across species. More importantly, rising evidence suggests their vital roles in gene regulation and association with diseases. As the fundamental effort toward elucidating their functions and mechanisms, several computational methods have been proposed to predict the circular structure from the primary sequence. Recently, advanced computational methods leverage deep learning to capture the relevant patterns from RNA sequences and model their interactions to facilitate the prediction. However, these methods fail to fully explore positional information of splice junctions and their deep interaction.

**Results:**

We present a robust end-to-end framework, Junction Encoder with Deep Interaction (JEDI), for circRNA prediction using only nucleotide sequences. JEDI first leverages the attention mechanism to encode each junction site based on deep bidirectional recurrent neural networks and then presents the novel cross-attention layer to model deep interaction among these sites for backsplicing. Finally, JEDI can not only predict circRNAs but also interpret relationships among splice sites to discover backsplicing hotspots within a gene region. Experiments demonstrate JEDI significantly outperforms state-of-the-art approaches in circRNA prediction on both isoform level and gene level. Moreover, JEDI also shows promising results on zero-shot backsplicing discovery, where none of the existing approaches can achieve.

**Availability and implementation:**

The implementation of our framework is available at https://github.com/hallogameboy/JEDI.

**Supplementary information:**

Supplementary data are available at *Bioinformatics* online.

## 1 Introduction

The ENCODE project has revealed the vital role of different forms of non-protein-coding RNAs. Among these types of RNAs, much attention has been placed on cataloging and studying the long non-coding RNAs (lncRNAs), due to their high relevancy to gene regulation and diseases (Derrien *et al.*, 2012; Ponting *et al.*, 2009). lncRNAs are typical of 200 bp to >100 kb in length (Wang *et al.*, 2020). As a particular type of lncRNA, endogenous circular RNA (circRNA) has recently received a tremendous amount of research highlights not only because of its circularity, but also its implications in a myriad of human diseases, such as cancer and Alzheimer’s disease (Dube *et al.*, 2019; Qu *et al.*, 2018). circRNA arises during the process of alternative splicing of protein-coding genes, where the 5′ end of an exon is covalently ligated to the 3′ end of the same exon or a downstream exon, forming a closed continuous loop structure. This mechanism is also known as ‘backsplicing’. The circular structure provides several beneficial properties over the linear RNAs. To be more specific, it can serve as templates for rolling circle amplification of RNAs (Boss and Arenz, 2020), rearrange the order of genetic information (Lasda and Parker, 2014), resistant to exonuclease-mediated degradation (Jeck *et al.*, 2013) and create a constraint on RNA folding (Lasda and Parker, 2014). Although the consensus of biological functions, mechanisms and biogenesis remains unclear for most circRNAs (Barrett and Salzman, 2016; Yu and Kuo, 2019), there are emerging evidence suggesting their roles in acting as sponges for microRNAs (Hansen *et al.*, 2013; Memczak *et al.*, 2013), RNA-binding protein competition (Ashwal-Fluss *et al.*, 2014) and inducing host gene transcription (Li *et al.*, 2015). Evidently, as a fundamental step to facilitate the exploration of circRNA, it is essential to have a high-throughput approach to identify the circRNAs.

Multiple factors can contribute to the formation of circRNAs. These factors include complementary sequences in flanking introns (Ivanov *et al.*, 2015), the presence of inverted repeats (Dubin *et al.*, 1995), number of Arthrobacter luteus (ALU) and tandem repeats (Jeck *et al.*, 2013) and single nucleotide polymorphism (SNP) density (Thomas and Sætrom, 2014). These factors, together with the evolutionary conservation and secondary structure of RNA molecules, have been considered as the discriminative features for circRNA identification. Several research efforts (Chen *et al.*, 2018; Pan and Xiong, 2015; Wang and Wang, 2017) have leveraged these features to train a conventional statistical learning model to distinguish circRNAs from other lncRNAs. These statistical learning algorithms include support vector machines (SVM), random forest (RF) and multi-kernel learning. However, methods along this line often require an extensive domain-specific feature engineering process. Moreover, the selected features may not provide sufficient coverage to characterize the backsplicing event.

Recently, the rising of deep learning architectures have been widely adopted as an alternative learning algorithm that can alleviate the inadequacy of conventional statistical learning methods. Specifically, these deep learning algorithms provide powerful functionality to process large-scale data and automatically extract useful features for object tasks (LeCun *et al.*, 2015). In the domain of circRNA prediction, the convolution neural network (CNN) is the architecture that has been widely explored to automatically learn the critical features for prediction, either from the primary sequence (Chaabane *et al.*, 2020; Wang and Wang, 2019) or secondary structure (Fiannaca *et al.*, 2017). Although CNN is capable of capturing relevant local patterns on gene sequences, positional information of the splice junctions and global context of each splice site cannot be recognized. One of these approaches (Chaabane *et al.*, 2020) attempts to address this issue by applying recurrent neural networks (RNNs) to learn sequential and contextual information; however, the essential knowledge, such as splice sites and junctions, are still ignored.

Understanding the properties of splice sites and their relationships is one of the keys to master RNA splicing and the formation of circRNAs because the splicing event can be considered as interaction among those splice sites. To fathom the relations between splice sites, circDeep (Chaabane *et al.*, 2020) explicitly analyzes the nucleotide sequences of two splice sites to predict the circRNAs. DeepCirCode (Wang and Wang, 2019) utilizes CNNs to model the flanking regions around two splice sites to identify if there is a backsplice event. However, all of the existing methods fail in modeling deep interaction among splice sites for circRNA prediction. For example, circDeep only measures shallow interaction among splice sites on the nucleotide level; DeepCirCode is limited to examine only a single pair of splice sites and lacks the capacity of modeling more complex relations among splice sites on multi-isoform genes. Hence, there is an immense gap to comprehensively understand the relationship between splice sites and their interaction regarding the formation of circRNAs.

In this article, we propose the framework of Junction Encoder with Deep Interaction (JEDI) to address the limitations in circRNA prediction. More precisely, we focus on predicting the existence of circRNAs from either the reference gene/isoform sequences or assembled transcript sequences by modeling splice sites and their deep interaction with deep learning techniques. First, the attentive junction encoders are presented to derive continuous embedding vectors for acceptor and donor splice sites based on their flanking regions around junctions. Based on the acceptor and donor embeddings, we propose the novel cross-attention layer to model deep interaction between acceptor and donor sites, thereby inferring cross-attentive embedding vectors. Finally, the attention mechanism is applied to determine acceptors and donors that are more important than other ones to predict if there is a circRNA. It is also important to note that the interpretability of the attention mechanism and the cross-attention layer enables JEDI to automatically discover backsplicing without training on any annotated backspliced sites.

Our contributions are 3-fold. First, to the best of our knowledge, this work is the first study to model the deep interaction among splice sites for circRNA prediction. The more profound understandings of the relationships among splice sites can intuitively benefit circRNA prediction in implying backsplicing. Second, we propose a robust and effective end-to-end framework, JEDI, to deal with both isoform-level and gene-level circRNA prediction based on the attention mechanism and the innovative cross-attention layer. More specifically, JEDI is capable of not only deriving appropriate representations from junction encoders but also routing the importance of forming circRNAs on different levels. Third, JEDI creates a new opportunity of transferring the knowledge from circRNA prediction to backsplicing discovery based on its extensive usage of attention mechanisms. Moreover, our approach can be utilized as a general and user-friendly detection tool to provide a robust estimated ranking for further validation. Extensive experiments on human circRNAs have demonstrated that JEDI significantly outperforms eight competitive baseline methods on both isoform level and gene level. The independent study on mouse circRNAs also indicates that JEDI is robust to transfer knowledge learned from human sequence to mouse for circRNA prediction. This phenomenon is supported by the observation of highly conserved circRNA across species (Barrett and Salzman, 2016; Jeck *et al.*, 2013; Suenkel *et al.*, 2020). In addition, we conduct the experiments to demonstrate that JEDI can automatically discover backspliced site pairs without any further annotations. Finally, an in-depth analysis of model hyperparameters and run-time presents the robustness and efficiency of JEDI.

## 2 Related work

Current works to discover circRNA can be divided into two categories: one relies on detecting backspliced junction reads from RNA-Seq data and the other examines features directly from transcript sequences.

The first category aims at detecting circRNA from expression data, specifically from RNA-Seq reads. It is mainly achieved by searching for chimeric reads that join the 3′ end to the upstream 5′ end with respect to a transcript sequence (Barrett and Salzman, 2016). Existing algorithms include *MapSplice* (Wang *et al.*, 2010), *CIRCexplorer* (Zhang *et al.*, 2014), *KNIFE* (Szabo *et al.*, 2015), *find-circ* (Memczak *et al.*, 2013) and *CIRI* (Gao *et al.*, 2015, 2018). These algorithms can be quite sensitive to the expression abundance, as circRNAs are often lowly expressed and fail to be captured with low sequencing coverage (Barrett and Salzman, 2016). In the comparison conducted by Hansen *et al.* (2016), the findings suggest dramatic differences among these algorithms in terms of sensitivity and specificity. Other caveats are reflected in long duration, high RAM usage and/or complicated pipeline.

The second category focuses on predicting the circRNA based on transcript sequences. Methods in this category leverage different features and learning algorithms to distinguish circRNA from other lncRNAs. *PredicircRNA* (Pan and Xiong, 2015) and *H-ELM* (Chen *et al.*, 2018) develop different strategies to extract discriminative features, and employ conventional statistical learning algorithms, i.e. multiple kernel learning for PredicircRNA and hierarchical extreme learning machine for H-ELM, to build a classifier. Statistical learning approaches require explicit feature engineering and selection. However, the extracted features are dedicated to specific facets of the sequence properties and present a limited coverage on the interaction information between the donor and acceptor sites. *circDeep* (Chaabane *et al.*, 2020) and *DeepCirCode* (Wang and Wang, 2019) are two pioneering methods that employ deep learning architectures to automatically learn complex patterns from the raw sequence without extensive feature engineering. circDeep uses CNNs with the bidirectional long short-term memory network (LSTM) to encode the entire sequence, whereas DeepCirCode uses CNNs with max-pooling to capture only the flanking sequences of the backsplicing sites. Although circDeep has claimed to be an end-to-end framework, it requires external resources and strategies to capture the reverse complement matching (RCM) features at the flanking sequence and the conservation level of the sequence. In addition, the RCM features only measure the match scores between sites on the nucleotide level, and neglect the complicated interaction between two sites. CNNs with max-pooling aim at preserving important local patterns within the flanking sequences. As a result, DeepCirCode fails to retain the positional information of nucleotides and their corresponding convoluted results.

Besides sequence information, a few conventional lncRNA prediction methods also present the potential of discovering circRNA through the secondary structure. *nRC* (Fiannaca *et al.*, 2017) extracts features from the secondary structures of non-coding RNAs and adopts CNNs framework to classify different types of non-coding RNA. *lncFinder* (Han *et al.*, 2019) integrates both the sequence composition and structural information as features and employs RFs. The learning process can be further optimized to predict different types of lncRNA. Nevertheless, none of these methods factor in the information specific to the formation of circRNAs, particularly the interaction information between splicing sites.

## 3 Materials and methods

In this section, we first formally define the objective of this article, and then present our proposed deep learning framework, JEDI, to predict circRNAs.

### 3.1 Preliminary and problem statement

The vocabulary of four nucleotides is denoted as V={A,C,G,T}. For a gene sequence *S*, $s[i\ldots j]\in V^{j-i+1}$ indicates the subsequence from the *i*th to the *j*th nucleotide of a sequence *S*. For a gene or an RNA isoform with the sequence *S*, $E(S)=\{(a_{i},d_{i})\}$ represents the given exons in the gene or the isoform, where *a_i_* and *d_i_* are the indices of the acceptor and donor junctions of the *i*th exon in *S*. Using only sequence information, the two goals of this work are listed as follows:

1. Isoform-level circRNA prediction: Given a gene sequence *S* and the splicing information of an isoform E(s), the goal is to identify whether this RNA isoform is a circRNA.
2. Gene-level circRNA prediction: Given a gene sequence *S* and all of its exon–intron boundaries E(S), this task aims at predicting if any of the junction pairs can backsplice to form a circRNA.

### 3.2 Framework overview

**Fig. 1.**
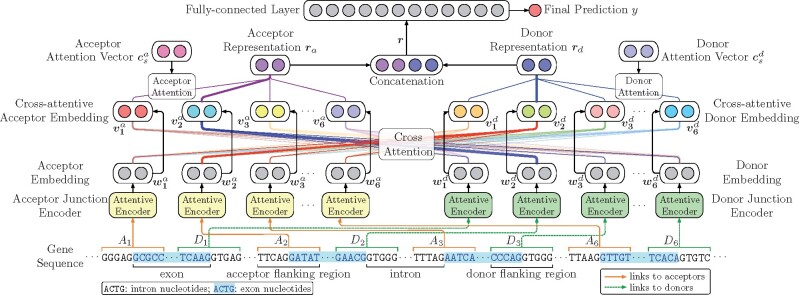
The schema of the proposed framework, JEDI, using the gene NM_001080433 with six exons as an example, where the second exon forms backsplicing. *A_i_* and *D_j_* represent the *i*th and *j*th potential acceptors and donors

Figure 1 illustrates the general schema of JEDI to predict circRNAs. Each acceptor *a_i_* and donor *d_j_* in the gene sequence are first represented by flanking regions *A_i_* and *D_i_* around exon–intron junctions. Two attentive junction encoders then derive embedding vectors of acceptors and donors, respectively. Based on the embedding vectors, we apply the cross-attention mechanism to consider deep interactions between acceptors and donors, thereby obtaining donor-aware acceptor embeddings and acceptor-aware donor embeddings. Finally, the attention mechanism is applied again to learn the provided acceptor and donor representations so that the prediction can be inferred by a fully connected layer based on the representations.

### 3.3 Attentive junction encoders

**Fig. 2.**
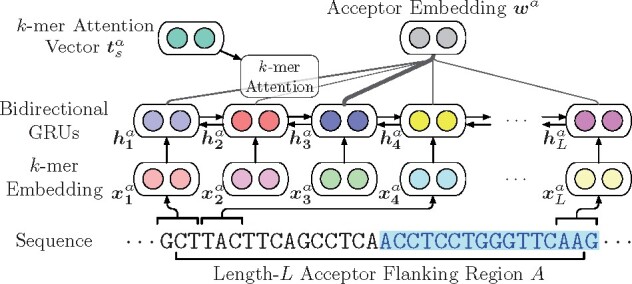
The illustration of the attentive encoder for acceptor junctions. Note that the donor junction encoder shares the same model structure with different model parameters

To represent the properties of acceptors and donors in the gene sequence *S*, we utilize the flanking regions around junctions to derive informative embedding vectors. Specifically, as shown in Figure 2, we propose attentive junction encoders using RNNs and the attention mechanism based on acceptor and donor flanking regions.

#### 3.3.1 Flanking regions as inputs

For each exon $\left(a_{i},d_{i})\in E(S\right)$, length *L* acceptor and donor flanking regions *A_i_* and *D_i_* can be computed as:

$$\begin{array}{c} A_{i}=\left[a_{i}-[\frac{L-1}{2}],\ldots ,a_{i}-1,a_{i},a_{i}+1,\ldots ,a_{i}+[\frac{L}{2}]\right],\\ D_{i}=\left[d_{i}-[\frac{L-1}{2}],\ldots ,d_{i}-1,d_{i},d_{i}+1,\ldots ,d_{i}+[\frac{L}{2}]\right], \end{array}$$

where $A_{i}[j]$ and $D_{i}[j]$ denote the *j*th positions on *S* for the flanking regions of the acceptor *a_i_* and the donor *d_i_*; the region length *L* is a tunable hyperparameter.

Suppose we are encoding an acceptor *a* and a donor *d* with the flanking regions *A* and *D* in the gene sequence *S* for the simplicity.

#### 3.3.2 *k*-mer embedding

To represent different positions in the sequence, we use *k*-mers as representations because *k*-mers are capable of preserving more complicated local contexts (Ju *et al.*, 2017). Each unique *k*-mer is then mapped to a continuous embedding vector as various deep learning approaches in bioinformatics (Chaabane *et al.*, 2020; Min *et al.*, 2017). Formally, for each position A[j] and D[j], the corresponding *k*-mer embedding vectors $x_{j}^{a}$ and $x_{j}^{d}$ can be derived as follows:

$$\begin{array}{c} x_{j}^{a}=F\left(S\left[A[j]-[\frac{K-1}{2}]\ldots A[j]+[\frac{K}{2}]\right]\right),\\ x_{j}^{d}=F\left(S\left[D[j]-[\frac{K-1}{2}]\ldots D[j]+[\frac{K}{2}]\right]\right), \end{array}$$

where $F(\cdot ):V^{K}\mapsto R^{l}$ is an embedding function mapping a length *K k*-mer to a *l*-dimensional continuous representation; the embedding dimension *l* and the *k*-mer length *K* are two model hyperparameters. Subsequently, *A* and *D* are represented by the corresponding *k*-mer embedding sequences, $x^{a}=[x_{1}^{a},\ldots ,x_{L}^{a}]$ and $x^{d}=[x_{1}^{d},\ldots ,x_{L}^{d}]$.

#### 3.3.3 Bidirectional RNNs

Based on *k*-mer embedding vectors, we apply bidirectional RNNs (BiRNNs) to learn the sequential properties in genes. The *k*-mer embedding sequences are scanned twice in both directions as forward and backward passes. During the forward pass, BiRNNs compute forward hidden states $\vec{h^{a}}$ and $\vec{h^{d}}$ as:

$$\vec{h^{a}}=[\vec{h_{1}^{a}},\ldots ,\vec{h_{L}^{a}}]\;\text{and}\;\vec{h^{d}}=[\vec{h_{1}^{d}},\ldots ,\vec{h_{L}^{d}}],$$

where $\vec{h_{j}^{a}}={\vec{\text{GRU}}}_{a}(\vec{h_{\mathrm{j-1}}^{a}},x_{j}^{a})$; $\vec{h_{j}^{d}}={\vec{\text{GRU}}}_{d}(\vec{h_{\mathrm{j-1}}^{d}},x_{j}^{d})$. $\vec{{\text{GRU}}_{a}}$ and $\vec{{\text{GRU}}_{d}}$ are gated recurrent units (GRUs) (Cho *et al.*, 2014) with different parameters for acceptors and donors, respectively. Note that we adopt GRUs instead of other RNN cells like LSTM (Hochreiter and Schmidhuber, 1997) because GRUs require fewer parameters (Jozefowicz *et al.*, 2015). Similarly, the backward pass reads the sequences in the opposite order, thereby calculating backward hidden states $\overset{\leftarrow }{h^{a}}$ and $\overset{\leftarrow }{h^{d}}$ as:

$$\overset{\leftarrow }{h^{a}}=[\overset{\leftarrow }{h_{1}^{a}},\ldots ,\overset{\leftarrow }{h_{L}^{a}}]\text{and}\;\overset{\leftarrow }{h^{d}}=[\overset{\leftarrow }{h_{1}^{d}},\ldots ,\overset{\leftarrow }{h_{L}^{d}}],$$

where $\overset{\leftarrow }{h_{j}^{a}}={\overset{\leftarrow }{\text{GRU}}}_{a}(\overset{\leftarrow }{h_{\mathrm{j+1}}^{a}},x_{j}^{a})$; $\overset{\leftarrow }{h_{j}^{d}}={\overset{\leftarrow }{\text{GRU}}}_{d}(\overset{\leftarrow }{h_{\mathrm{j+1}}^{d}},x_{j}^{d})$. To model *k*-mers with context information, we concatenate forward and backward hidden states as the hidden representations of *k*-mers in *A* and *D* as:

$$h^{a}=[h_{1}^{a},\ldots ,h_{L}^{a}]\;\text{and}\;h^{d}=[h_{1}^{d},\ldots ,h_{L}^{d}],$$

where $h_{j}^{a}=[\vec{h_{j}^{a}}$; $\overset{\leftarrow }{h_{j}^{a}}]$; $h_{j}^{d}=[\vec{h_{j}^{d}};\overset{\leftarrow }{h_{j}^{d}}]$.

#### 3.3.4 *k*-mer attention

Since different *k*-mers can have unequal importance for representing the properties of splice sites, we introduce the attention mechanism (Bahdanau *et al.*, 2015) to identify and aggregate the hidden representations of *k*-mers that are more important than others. The motivation of the attention mechanism is to learn a computational function for automatically estimating the importance score of each item so that the ultimate representation can focus on items that are more significant. More precisely, the importance scores of representations $h_{j}^{a}$ and $h_{k}^{d}$ can be estimated by the *k*-mer attention vectors $t_{s}^{a}$ and $t_{s}^{d}$ as:

$$\alpha_{j}^{a}=\frac{\;\text{exp}(t_{j}^{a}{}^{T}t_{s}^{a})}{\sum_{j\prime }\;\text{exp}\;(t_{j\prime }^{a}{}^{T}t_{s}^{a})}\;\text{and}\;\alpha_{j}^{d}=\frac{\;\text{exp}(t_{j}^{d}{}^{T}t_{s}^{d})}{\sum_{j\prime }\;\text{exp}\;(t_{j\prime }^{d}{}^{T}t_{s}^{d})},$$

where $t_{j}^{a}=\tanh(F_{t}^{a}(h_{j}^{a}))$; $t_{j}^{d}=\tanh(F_{t}^{d}(h_{j}^{d}))$; $F_{t}^{a}(\cdot )$ and $F_{t}^{d}(\cdot )$ are fully connected layers. tanh(·) is the activation function for the convenience of similarity computation. The importance scores are first measured by the inner-products to the *k*-mer attention vectors and then normalized by a softmax function over the scores of all *k*-mers. Note that the *k*-mer attention vectors $t_{s}^{a}$ and $t_{s}^{d}$ are learnable and updated during optimization as model parameters. Finally, the acceptor embedding $w^{a}$ of *A* and the donor embedding $w^{d}$ of *D* can be derived by aggregating the hidden representations of *k*-mers weighted by their learned importance scores as:

$$w^{a}=\sum_{j}\alpha_{j}^{a}\cdot h_{j}^{a}\text{and}\;w^{d}=\sum_{j}\alpha_{j}^{d}\cdot h_{j}^{d}.$$

### 3.4 Cross-attention for modeling deep interaction

Modeling interactions among splice sites is essential for circRNA prediction because backsplices occur when the donors prefer the upstream acceptors over the downstream ones. Inspired by recent successes in natural language processing (Hao *et al.*, 2017) and computer vision (Lee *et al.*, 2018), we propose the cross-attention layer to learn deep interaction between acceptors and donors.

#### 3.4.1 Cross-attention layer

For acceptors, the cross-attention layer aims at deriving cross-attentive acceptor embeddings that not only represent the acceptor sites and their flanking regions but also preserve the knowledge of relevant donors from donor embeddings. Similarly, the cross-attentive donor embeddings are simultaneously obtained for donors. To directly model relations between embeddings, we adopt the dot-product attention mechanism (Vaswani *et al.*, 2017) for the cross-attention layer. For each acceptor embedding $w_{i}^{a}$, the relevance of a donor embedding $w_{j}^{d}$ can be computed by a dot-product $w_{i}^{a}{}^{T}w_{j}^{d}$ so that the attention weights $\beta_{i,j}^{a}$ can be calculated with a softmax function over all donors. Likewise, the attention weights $\beta_{j,i}^{d}$ for each donor embedding $w_{j}^{d}$ can also be measured by dot-products to the acceptor embeddings. Stated formally, we have:

$$\beta_{i,j}^{a}=\frac{\;\text{exp}(w_{i}^{a}{}^{T}w_{j}^{d})}{\sum_{j\prime }\;\text{exp}\;(w_{i}^{a}{}^{T}w_{j\prime }^{d})}\text{and}\beta_{j,i}^{d}=\frac{\;\text{exp}(w_{j}^{d}{}^{T}w_{i}^{a})}{\sum_{i\prime }\;\text{exp}\;(w_{j}^{d}{}^{T}w_{i\prime }^{d})}.$$

Therefore, the cross-attentive embeddings of acceptors and donors can then be derived by aggregations based on the attention weights as:

$$v_{i}^{a}=\sum_{j}\beta_{i,j}^{a}\cdot w_{j}^{d}\;\text{and}\;v_{j}^{d}=\sum_{i}\beta_{j,i}^{d}\cdot w_{i}^{a}.$$

Note that we do not utilize the multi-head attention mechanism (Vaswani *et al.*, 2017) because it requires much more massive training data to learn multiple projection matrices. As shown in Section 4, the vanilla dot-product attention is sufficient to obtain satisfactory predictions with significant improvements over baselines.

### 3.5 circRNA prediction

To predict circRNAs, we apply the attention mechanism (Bahdanau *et al.*, 2015) again to aggregate cross-attentive acceptor and donor embeddings into an acceptor representation and a donor representation as ultimate features to predict circRNAs.

#### 3.5.1 Acceptor and donor attention

Although the cross-attention layer provides information cross-attentive embeddings for all acceptors and donors, most of the splice sites can be irrelevant to backsplicing. To tackle this issue, we present the acceptor and donor attention to identify splice sites that are more important than other ones. Similar to *k*-mer attention, the importance scores of cross-attentive embeddings for acceptors and donors can be computed as:

$$\gamma_{i}^{a}=\frac{\;\text{exp}(c_{i}^{a}{}^{T}c_{s}^{a})}{\sum_{i\prime }\;\text{exp}\;(c_{i\prime }^{a}{}^{T}c_{s}^{a})}\;\text{and}\;\gamma_{j}^{d}=\frac{\;\text{exp}(c_{j}^{d}{}^{T}c_{s}^{d})}{\sum_{j\prime }\;\text{exp}\;(c_{j\prime }^{d}{}^{T}c_{s}^{d})},$$

where $c_{i}^{a}=\tanh(F_{c}^{a}(v_{i}^{a}))$; $c_{j}^{d}=\tanh(F_{c}^{d}(v_{j}^{d}))$; $F_{c}^{a}(\cdot )$ and $F_{c}^{d}(\cdot )$ are fully connected layers. Subsequently, the acceptor and donor representations $r_{a}$ and $r_{d}$ can be derived based on the attention weights of cross-attentive embeddings as:

$$r_{a}=\sum_{i}\gamma_{i}^{a}\cdot v_{i}^{a}\;\text{and}\;r_{d}=\sum_{i}\gamma_{i}^{d}\cdot v_{i}^{d}.$$

#### 3.5.2 Prediction as binary classification

Here, we treat circRNA prediction as a binary classification task. More specifically, we estimate a probabilistic score $\hat{y}$ to approximate the probability of existing circRNA. The ultimate features ***r*** for machine learning are provided by concatenating the acceptor and donor representations as $r=[r_{a};r_{d}]$. Finally, the probabilistic score $\hat{y}$ can be computed by a sigmoid function with a fully connected layer as follows:

$$\hat{y}=\sigma (F_{p}(\text{ReLU}(F_{r}(r)))),$$

where $F_{p}(\cdot )$ and $F_{r}(\cdot )$ are fully connected layers; ReLU(·) is the activation function for the hidden layer (Glorot *et al.*, 2011); σ(·) is the logistic sigmoid function (Han and Moraga, 1995). The binary prediction can be further generated by a binary indicator function as $1\left(\hat{y}>0.5\right)$.

### 3.6 Learning and optimization

To solve circRNA prediction as a binary classification problem, JEDI is optimized with a binary cross-entropy (Hinton and Salakhutdinov, 2006). Formally, the loss function for optimization can be written as follows:

$$\text{Loss}=\frac{1}{N}\sum_{i=1}^{N}\left[y_{i}\;\text{log}(\hat{y_{i}})+(1-y_{i})\;\text{log}(1-\hat{y_{i}})\right]+\lambda ||\theta |{|}_{2},$$

where *N* is the number of training gene sequences; *y_i_* is a binary indicator demonstrating whether the *i*th training sequence exists a circRNA; $\hat{y_{i}}$ is the approximated probabilistic score for the *i*th training gene sequence; *λ* is the L2-regularization weight for the set of model parameters *θ*.

### 3.7 Remarks on the interpretability of JEDI

The usage of attention mechanisms is one of the most essential keys in JEDI, including the donor and acceptor attention, the cross-attention layer and the *k*-mer attention in junction encoders. In addition to choosing important information to optimize the objective, one of the most significant benefits of using attention mechanisms is the interpretability.

#### 3.7.1 Application: zero-shot backsplicing discovery

For circRNAs, the attention weights can become interpretable hints for discovering backsplicing without training on the annotated backspliced sites. For example, when the model is optimized for accurately predicting circRNAs, the weights of donor attention are reformed to denote the important and relevant donors, which are preferred for the upstream acceptors to backsplice. In other words, the probabilistic attention weight $\gamma_{j}^{d}$ for each donor *d_j_* can be interpreted as the probability of being a backsplice donor site as:

$$P(d_{j})=\gamma_{j}^{d},$$

where the softmax function guarantees $\sum_{j}P(d_{j})=1$. Similarly, the attention weight $\beta_{j,i}^{d}$ of each acceptor *a_i_* for deriving the cross-attentive embedding of the donor *d_j_* can be explained as the conditional probability of being selected as the backsplice acceptor site from the donor *d_j_* as:

$$P(a_{i}|d_{j})=\beta_{j,i}^{d},$$

where we also have the probabilistic property $\forall j:\sum_{i}\beta_{j,i}^{d}=1$ from the softmax function. Based on the above interpretations, for any pair of a donor *d_j_* and an acceptor *a_i_*, the probability of forming a backsplice can be approximated by decomposing the joint probability $P(d_{j},a_{i})$ as:

$$P(d_{j},a_{i})=P(d_{j})P(a_{i}|d_{j})=\gamma_{j}^{d}\beta_{j,i}^{d}.$$

Therefore, without any training backsplice site annotation as zero-shot learning (Socher *et al.*, 2013), we can transfer the knowledge in the training data for circRNA prediction to discover potential backsplice sites by ranking the pairs of acceptors and donors according to $P(d_{j},a_{i})$. Particularly, the interpretations can be also aligned with the process of RNA splicing, bringing more biological insights into JEDI. In Section 4.6, we further conduct experiments to demonstrate that JEDI is capable of addressing the task of zero-shot backsplicing discovery.

## 4 Experiments

In this section, we conduct extensive experiments on benchmark datasets for two tasks and in-depth analysis to verify the performance and robustness of the proposed framework, JEDI.

### 4.1 Datasets

#### 4.1.1 Human circRNA

We use the benchmark dataset generated by Chaabane *et al.* (2020). The positive data generation follows a similar setting as described in Pan and Xiong (2015) to derive 31 939 isoforms of human circRNAs covering a diverse range of tissues and cell types from the circRNADb database (Chen *et al.*, 2016). The negative set is composed of other lncRNAs, such as processed transcripts, anti-sense, sense intronic and sense overlapping. It is constructed based on the annotation provided by GENCODE v19 (Frankish *et al.*, 2019) with strong evidence. Specifically, only the experimentally validated or manually annotated transcripts are considered, resulting in 19 683 negative isoforms. To avoid information leaks through training and evaluating on paralogous genes, we group isoforms into the same cluster if they come from the same gene or duplicated genes. The duplicated gene information is retrieved from the Duplicated Genes Database (Ouedraogo *et al.*, 2012). Combining both the positive and negative cases, these 51 622 isoforms are grouped into 23 674 clusters. The clusters are divided into five parts to conduct 5-fold cross-validation. The sequences of all positive and negative cases are based on hg19.

#### 4.1.2 Mouse circRNA on isoform level

The mouse circRNAs are obtained through *circbase* (Glažar *et al.*, 2014), which contains public circRNA datasets for several species reported in literature. There are 1903 mouse circRNAs. Using the annotation provided by GENCODE vM1, we randomly select other lincRNAs, generating 1522 negative cases. The sequences of all positive and negative cases are based on mm9.

### 4.2 Experimental settings

#### 4.2.1 Baseline methods

To evaluate the performance of JEDI, we compare with eight competitive baseline methods, including circDeep (Chaabane *et al.*, 2020), PredcircRNA (Pan and Xiong, 2015), DeepCirCode (Wang and Wang, 2019), nRC (Fiannaca *et al.*, 2017), SVM, RF, attentive-CNN (Att-CNN) and attentive-RNN (Att-RNN). Specifically, circDeep and PredcircRNA are the state-of-the-art circRNA prediction methods. DeepCirCode originally takes individual splice site pairs for backsplicing prediction, which is another research problem, and leads to an enormous number of false alarms in our problem settings. To conduct fair comparisons, we modify DeepCirCode by extending the inputs to all sites and aggregating CNN representations for acceptors and donors with two max-pooling layers before applying its model structure. nRC represents lncRNA classification methods that are compatible to solve circRNA prediction as a sequence classification problem. SVM and RF apply conventional statistical learning frameworks with the compositional *k*-mer features proposed by Wang and Wang (2017) for backsplicing prediction. Attentive CNN and RNN as popular deep learning approaches utilize CNNs and RNNs with the attention mechanism (Bahdanau *et al.*, 2015) for sequence modeling, thereby predicting circRNAs based on a fully connected hidden layer with the ReLU activation function (Glorot *et al.*, 2011). Note that we do not compare with CIRCexplorer2 (Zhang *et al.*, 2016) and CIRI (Gao *et al.*, 2015) because they aim at aligning the sequencing reads to known circRNAs, and performing *de novo* assembly of novo circRNAs, which is a completely different approach than our proposed method.

#### 4.2.2 Evaluation metrics and protocol

Six conventional binary classification metrics are selected as the evaluation metrics for both tasks, including the overall accuracy (Acc), precision (Prec), sensitivity (Sens), specificity (Spec), F1-score as well as Matthew correlation coefficient (MCC) and the area under the receiver operating characteristic (ROC) curve (AUC) on positive cases. For all metrics, the higher metric scores indicate more satisfactory performance. We conduct a 5-fold cross-validation for evaluation on both isoform-level and gene-level circRNA prediction. Specifically, for each task, the data are randomly shuffled and evenly partitioned into five non-overlapping subsets. In the five folds of experiments, each subset has a chance to be considered as the testing data for assessing the model trained by the remaining four subsets, thereby ensuring an unbiased and fair evaluation. Finally, we evaluate the methods by aggregating the scores over the 5-fold experiments for each metric.

#### 4.2.3 Implementation details

Our approach, JEDI, is implemented in Tensorflow (Abadi *et al.*, 2016) and released in GitHub as shown in Abstract. The AMSGrad optimizer (Reddi *et al.*, 2018) is adopted to optimize the model parameters with a learning rate $\eta =10^{-3}$, exponential decay rates $\beta_{1}=0.9$ and $\beta_{2}=0.999$, a batch size 64, and an L2-regularization weight $\lambda =10^{-3}$. As the hyperparameters of JEDI, the *k*-mer size *K* and the number of dimensions *l* for *k*-mer embeddings are set to 3 and 128. We set the length of flanking regions *L* to 4. The hidden state size of GRUs for both directions in junction encoders is 128. The size of all attention vectors is set to 16. The number of units in the fully connected hidden layer $F_{r}(\cdot )$ for circRNA prediction is 128. The model parameters are trained until the convergence for each fold in cross-validation. For the baseline methods, the experiments for circDeep, PredcircRNA and nRC are carried out according to the publicly available implementations released by the authors of original papers. SVM and RF are implemented in Python with the scikit-learn library (Pedregosa *et al.*, 2011). As for deep learning approaches, DeepCirCode, Att-CNN and Attentive-RNN are implemented in Tensorflow, which is the same as our proposed JEDI. For all methods, we conduct parameter fine-tuning for fair comparisons. All of the experiments are also equitably conducted on a computational server with one NVIDIA Tesla V100 GPU and one 20-core Intel Xeon CPU E5-2698 v4 @ 2.20 GHz.

### 4.3 Isoform-level circRNA prediction

Table 1 shows the performance of all methods for isoform-level circRNA prediction. Among the baseline methods, circDeep as the state-of-the-art approach and DeepCirCode considering junctions perform the best. It is because circDeep explicitly accounts for the reverse complimentary sequence matches in flanking regions of the junctions, and DeepCirCode models the flanking regions with deep learning. Consistent with the previous study (Chaabane *et al.*, 2020), PredcircRNA performs worse than circDeep. With compositional *k*-mer-based features designed for backsplicing prediction, SVM and RF surprisingly outperform PredicircRNA by 11.13% and 16.14% in accuracy. It not only shows that the *k*-mers are universally beneficial across different tasks but also emphasizes the rationality of using *k*-mers for junction encoders in JEDI. As an lncRNA classification method, nRC also shows its potential for circRNA prediction with a 15.37% improvement over PredcircRNA in accuracy. Although Att-CNN and Att-RNN utilize the attention mechanism, they can only model the whole sequences and present limited performance without any knowledge of junctions. As our proposed approach, JEDI significantly outperforms all of the baseline methods across all evaluation metrics. Particularly, JEDI achieves 9.80% and 7.90% improvements over DeepCirCode in accuracy and F1-score, respectively. The experimental results have demonstrated the effectiveness of junction encoders and the cross-attention layer that models deep interaction among splice sites.

**Table 1.** Evaluation of isoform-level circular RNA prediction based on the 5-fold cross-validation

Method	Accuracy	Precision	Sensitivity	Specificity	F1-score	MCC	AUC
SVM	0.7279 ± 0.0686	0.7479 ± 0.0970	0.8932 ± 0.1075	0.4526 ± 0.3413	0.8042 ± 0.0260	0.4031 ± 0.1784	0.6729 ± 0.1203
RF	0.7607 ± 0.0084	0.7764 ± 0.0123	0.8610 ± 0.0077	0.5982 ± 0.0075	0.8165 ± 0.0095	0.4804 ± 0.0115	0.7296 ± 0.0053
Att-CNN	0.7519 ± 0.0069	0.7739 ± 0.0257	0.8529 ± 0.0391	0.5872 ± 0.0527	0.8105 ± 0.0080	0.4612 ± 0.0165	0.7200 ± 0.0097
Att-RNN	0.7638 ± 0.0075	0.7773 ± 0.0161	0.8582 ± 0.0345	0.6171 ± 0.0505	0.8152 ± 0.0094	0.4960 ± 0.0134	0.7377 ± 0.0105
nRC	0.7557 ± 0.0115	0.7844 ± 0.0389	0.8410 ± 0.0597	0.6193 ± 0.0998	0.8094 ± 0.0118	0.4781 ± 0.0279	0.8280 ± 0.0091
PredcircRNA	0.6550 ± 0.0076	0.6977 ± 0.0137	0.5949 ± 0.0070	0.7202 ± 0.0120	0.6422 ± 0.0091	0.3169 ± 0.0164	0.5882 ± 0.0102
circDeep	0.8748 ± 0.0102	0.9393 ± 0.0134	0.8161 ± 0.0217	0.9407 ± 0.0138	0.8732 ± 0.0111	0.7584 ± 0.0186	0.7395 ± 0.0132
DeepCirCode	0.8997 ± 0.0039	0.9353 ± 0.0228	0.9021 ± 0.0248	0.8967 ± 0.0383	0.9179 ± 0.0040	0.7914 ± 0.0073	0.8994 ± 0.0077
JEDI	0.9878 ± 0.0007	0.9906 ± 0.0030	0.9906 ± 0.0032	0.9836 ± 0.0038	0.9904 ± 0.0009	0.9742 ± 0.0014	0.9872 ± 0.0009

*Note*: We report the mean and standard deviation for each metric.

### 4.4 Gene-level circRNA prediction

We further evaluate all methods on gene-level circRNA prediction. Note that this task is more difficult than the isoform-level prediction because each junction can be a backsplice site. Since a full gene sequence can encode for multiple isoforms, there can be multiple site pairs forming backsplices for different isoforms. Consequently, models cannot learn from absolute positions for circRNA prediction. As shown in Table 2, all methods deliver worse performance than the results in isoform-level circRNA prediction. Notably, the evaluation metrics have demonstrated a similar trend as shown in Table 1. DeepCirCode and circDeep are still the best baseline methods, showing the robustness of exploiting the knowledge about splice junctions. SVM, RF and nRC still outperform PredicircRNA by at least 15.08% in accuracy. Att-CNN and Att-RNN using the attention mechanism still fail to obtain extraordinary performance because they are unaware of junction information, which is essential for backsplicing events. In this more difficult task, JEDI consistently surpasses all of the baseline methods across all evaluation metrics. For instance, JEDI beats DeepCirCode by 11.94% and 11.75% in accuracy and F1-score, respectively. The experimental results further reveal that our proposed JEDI is capable of tackling different scenarios of circRNA prediction with consistently satisfactory predictions.

**Table 2.** Evaluation of gene-level circular RNA prediction based on the 5-fold cross-validation

Method	Accuracy	Precision	Sensitivity	Specificity	F1-score	MCC	AUC
SVM	0.7121 ± 0.0560	0.8346 ± 0.0596	0.5836 ± 0.1958	0.8534 ± 0.1067	0.6652 ± 0.1212	0.4662 ± 0.0719	0.7185 ± 0.0504
RF	0.7324 ± 0.0060	0.7035 ± 0.0097	0.8499 ± 0.0041	0.6018 ± 0.0131	0.7697 ± 0.0050	0.4688 ± 0.0105	0.7259 ± 0.0058
Att-CNN	0.7246 ± 0.0051	0.7555 ± 0.0275	0.8302 ± 0.0454	0.5519 ± 0.0624	0.7898 ± 0.0088	0.4006 ± 0.0071	0.6910 ± 0.0091
Att-RNN	0.7300 ± 0.0076	0.7565 ± 0.0246	0.8340 ± 0.0407	0.5639 ± 0.0546	0.7923 ± 0.0086	0.4164 ± 0.0153	0.6989 ± 0.0099
nRC	0.7290 ± 0.0086	0.7378 ± 0.0329	0.7592 ± 0.0594	0.6961 ± 0.0663	0.7461 ± 0.0154	0.4591 ± 0.0190	0.8013 ± 0.0076
PredcircRNA	0.6188 ± 0.0033	0.6594 ± 0.0085	0.5732 ± 0.0085	0.6696 ± 0.0117	0.6132 ± 0.0049	0.2433 ± 0.0077	0.6085 ± 0.0314
circDeep	0.8387 ± 0.0066	0.8778 ± 0.0135	0.8063 ± 0.0087	0.8749 ± 0.0133	0.8404 ± 0.0073	0.6806 ± 0.0138	0.7522 ± 0.0107
DeepCirCode	0.8629 ± 0.0215	0.8940 ± 0.0268	0.8424 ± 0.0606	0.8860 ± 0.0374	0.8659 ± 0.0265	0.7296 ± 0.0377	0.8642 ± 0.0198
JEDI	0.9659 ± 0.0059	0.9670 ± 0.0075	0.9685 ± 0.0166	0.9630 ± 0.0095	0.9676 ± 0.0057	0.9318 ± 0.0119	0.9657 ± 0.0054

*Note*: We report the mean and standard deviation for each metric.

### 4.5 Independent study on mouse circRNAs

To demonstrate the robustness of JEDI, we conduct an independent study on the dataset of mouse circRNAs. Previous studies have shown that circRNAs are evolutionarily conserved (Barrett and Salzman, 2016; Jeck *et al.*, 2013; Suenkel *et al.*, 2020), and thus we evaluate the potential of predicting the circRNAs across different species. More precisely, we train each method using the human dataset on isoform level, thereby predicting the circRNAs on the mouse dataset. Note that some of the required features for PredcircRNA are missing on the mouse datasets. In addition to this, PredicircRNA perform the worst in other experiments. For these reasons, we exclude PredcircRNA from this study. Table 3 presents the experimental results of the independent study. Compared to the experiments conducted on the same species as shown in Table 1, most of the deep learning methods have slightly lower performance because they are specifically optimized for human data; SVM and RF have similar performance in the independent study probably because *k*-mer features are simpler and more general to different species. Interestingly, the accuracy of circDeep significantly drops in the study. It is likely due to the fact that circDeep heavily pre-trains the sequence modeling on human data with the serious over-fitting phenomenon. As a result, our proposed JEDI still outperforms all of the baseline methods. It demonstrates that JEDI is robust across the datasets of different species.

**Table 3.** Independent study of isoform-level circular RNA prediction for mouse circRNAs based on the models trained on human circRNAs

Method	Acc	Prec	Sens	Spec	F1	MCC	AUC
SVM	0.7328	0.7742	0.8108	0.6011	0.7921	0.4196	0.7059
RF	0.7186	0.7393	0.8523	0.4929	0.7918	0.3733	0.6726
Att-CNN	0.7264	0.7452	0.7957	0.6330	0.7696	0.4352	0.7143
Att-RNN	0.7030	0.7189	0.7930	0.5816	0.7541	0.3844	0.6873
PredicircRNA	0.5696	0.6218	0.5056	0.6437	0.5577	0.1501	0.6067
nRC	0.7410	0.7662	0.8455	0.5647	0.8039	0.4298	0.8097
circDeep	0.6140	0.7495	0.6982	0.7509	0.7229	0.4491	0.7669
DeepCirCode	0.8129	0.9271	0.7620	0.8989	0.8365	0.6392	0.8304
JEDI	0.8654	0.9074	0.8749	0.8493	0.8909	0.7162	0.8621

### 4.6 Zero-shot backsplicing discovery

**Fig. 3.**
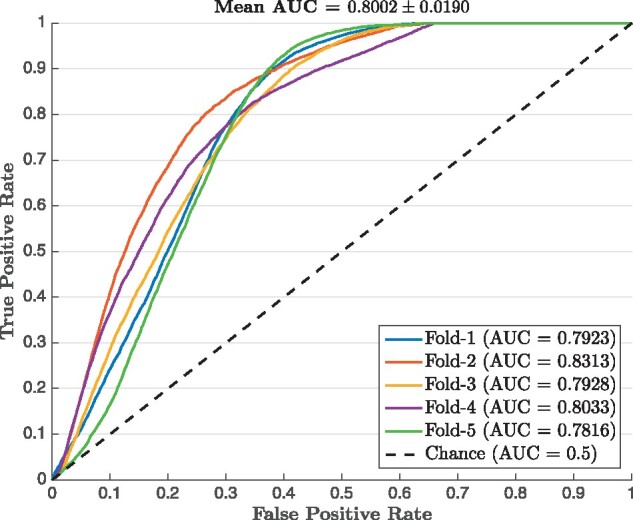
The ROC curves for zero-shot backsplicing discovery based on the 5-fold cross-validation and JEDI trained for gene-level circular RNA prediction

As mentioned in Section 3.7, the interpretability of the attention mechanisms and the cross-attention layer enables JEDI to achieve zero-shot backsplicing discovery. To evaluate the performance of zero-shot backsplicing, we compute the probabilistic score $P(d_{j},a_{i})$ using the attention weights $\gamma_{j}^{d}$ and $\beta_{j,i}^{d}$, thereby indicating the likelihood of forming a backsplice for each pair of a candidate donor *d_j_* and a candidate acceptor *a_i_*. Hence, we can simply evaluate the probabilistic scores with the ROC curve and the AUC. Note that here we still apply 5-fold cross-validation for experiments based on the gene-level human circRNA dataset. Since none of the existing methods can address the task of zero-shot backsplicing prediction, we compare with random guessing, which is equivalent to the chance line in ROCs with an AUC score of 0.5. Figure 3 depicts the ROC curves with AUC scores over five folds of experiments. The results show that the backspliced site pairs discovered by JEDI are effective with an average AUC score of 0.8002. In addition, JEDI is also robust in this task with a small standard deviation of AUC scores. Since the cross-attention layer is a major contribution in JEDI, we conduct another study to analyze how donor and acceptor embeddings interact with each other in Supplementary Section S1.

### 4.7 Analysis and discussions

In this section, we first discuss the impacts of hyperparameters for JEDI and then conduct the run-time analysis for all methods to verify the model efficiency of JEDI. Note that, for hyperparameter analysis, we adjust the target hyperparameter while other hyperparameters are fixed as the values utilized in the experiments as mentioned in Section 4.2.

#### 4.7.1 Length of flanking regions *L*

**Fig. 4.**
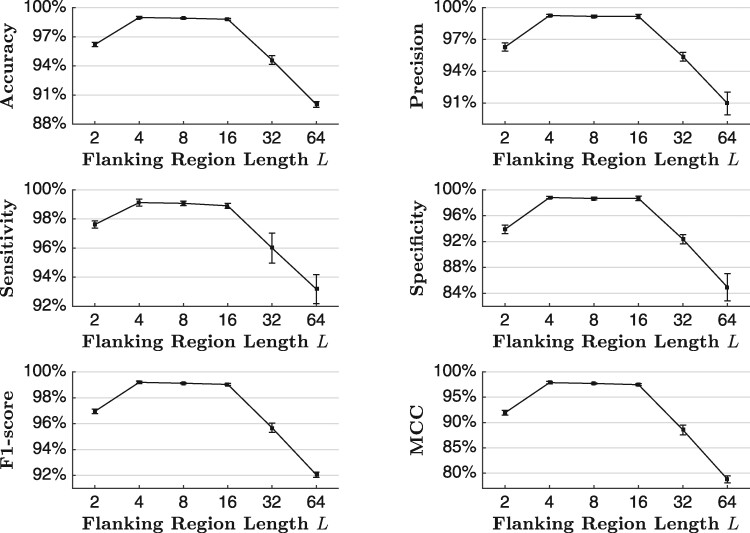
The isoform-level circular RNA prediction performance of JEDI with different flanking region lengths *L* based on the 5-fold cross-validation. We report the mean for each metric and apply error bars to indicate standard deviations

The flanking region length *L* for junction encoders plays an important role in JEDI to represent splice sites. Figure 4 illustrates the circRNA prediction performance of JEDI over different flanking region lengths. For all evaluation metrics, the performance slightly improves when *L* increases to 4. However, the performance significantly drops when L≥32. It shows that nucleotides nearer to junctions are more important than other ones for predicting backsplicing. This result is also consistent with previous studies on RNA splicing (Ohshima and Gotoh, 1987). Moreover, circRNAs tend to contain fewer nucleotides than other transcripts from the same gene (Jeck and Sharpless, 2014), so excessive and redundant information could only lead to noises and lower the prediction performance.

#### 4.7.2 Size of *k*-mers *K*

**Fig. 5.**
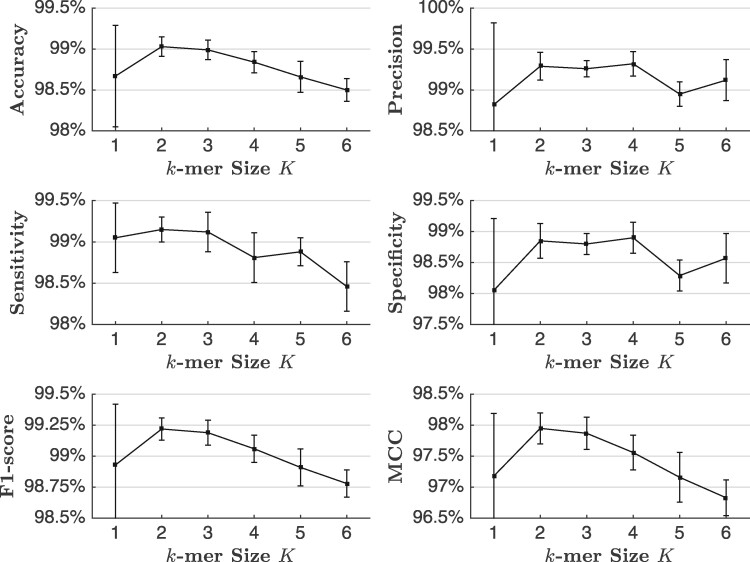
The isoform-level circular RNA prediction performance of JEDI with different *k*-mer sizes *K* based on the 5-fold cross-validation. We report the mean for each metric and apply error bars to indicate standard deviations

The derivation of *k*-mers is crucial for JEDI because JEDI treats *k*-mers as the fundamental inputs over gene sequences. Figure 5 shows how the size of *k*-mers affects the prediction performance. JEDI performs the best with 2- and 3-mers when the performance gets worse with longer or shorter *k*-mers. It could be because a small *k*-mer size makes *k*-mers less significant for representations. In addition, the embedding space of long *k*-mers could be too enormous for JEDI to learn with limited training data. It is also worthwhile to mention that 1-mers lead to much higher standard deviations because of their low significance induces high instability and sensitive embeddings during the learning process. This finding is also consistent with previous studies (Sacan and Toroslu, 2008).

In addition to the flanking region length *L* and the *k*-mer size *K*, we also conduct the analysis to study how the embedding dimension *l* affects the performance in Supplementary Section S2.

#### 4.7.3 Run-time analysis

To verify the efficiency of JEDI, we conduct the run-time analysis for all methods in our experiments based on the task of isoform-level circRNA prediction. For fair comparisons, all methods can access the same computational resources. Note that we only consider the time in training and testing. The run-time of feature extraction and disk I/O are ignored because the features can be pre-processed. Disk I/O can be affected by many factors that are irrelevant to methods, such as I/O scheduling in operating systems. As shown in Table 4, JEDI is efficient and averagely needs only <3 min because it only focuses on junctions and flanking regions. Similarly, DeepCirCode, which is also a junction-based deep learning method, has comparable execution time to JEDI. In contrast, Att-CNN and Att-RNN are relatively inefficient because they scan the whole sequences in every training batch, where Att-RNN with non-parallelizable recurrent units is slower. Although nRC reads the whole sequences, it runs faster than some attention-based methods because of its simpler model structure. SVM, RF and PredcircRNA are the most efficient because they apply straightforward statistical machine learning frameworks for training. As a side note, the feature extraction of PredcircRNA is extremely expensive in execution time and averagely costs more than 28 h to extract multi-facet features in our experiments. circDeep is the most inefficient in our experiments because it consists of many time-consuming components, such as embedding and LSTM pre-training.

**Table 4.** Run-time analysis on isoform-level circular RNA prediction in seconds (s), minutes (min) and hours (h), based on the 5-fold cross-validation

Method	Time	Method	Time	Method	Time
SVM	28.76 s	Att-CNN	13.35 min	circDeep	>24 h
RF	21.03 s	Att-RNN	51.53 min	DeepCirCode	3.80 min
nRC	4.07 min	PredcircRNA	43.66s	JEDI	2.75 min

*Note*: We report the mean of the training time (over five folds).

## 5 Conclusions

In this article, we propose a novel end-to-end deep learning approach for circRNA prediction by learning to appropriately model splice sites with flanking regions around junctions and studying the deep relationships among these sites. The attentive junction encoders are first introduced to represent each splice site, and the innovative cross-attention layer is proposed to learn the deep interaction among splice sites. Moreover, JEDI is capable of discovering backspliced site pairs without training on annotated site pairs. The experimental results demonstrate that JEDI is effective and robust in circRNA prediction on different data levels and across different species. Most importantly, the backspliced site pairs discovered by JEDI are promising as they designate the hotspots for circRNAs formation. The reasons and insights for these observations and discoveries can be concluded as follows: (i) JEDI only models valuable and essential flanking regions around the junctions of splice sites, thereby discarding irrelevant and redundant information for circRNA prediction; (ii) the properties of splice sites and essential information for forming circRNAs can be well-preserved by junction encoders; and (iii) the attention mechanisms and the cross-attention layer provide intuitive and interpretable hints to implicitly model the backsplicing events as demonstrated in the experiments. Due to data limitation, we are only able to examine the effectiveness of transferring the learned knowledge between humans and mice. As a future direction, we plan to experiment with more species when more data are available. Additionally, we also plan on exploring the potential to extend JEDI to support circRNA prediction from sequencing reads.

## Acknowledgement

We thank all reviewers for their constructive comments and valuable suggestions.

## Funding

This work was partially supported by the National Science Foundation [NSF-DGE-1829071, NSF-IIS-2031187] and the National Institutes of Health [NIH-R35-HL135772].

*Conflict of Interest*: none declared.

## Supplementary Material

btab288_Supplementary_Data
