# Supplementary material for: JEDI: circular RNA prediction based on junction encoders and deep interaction among splice sites
Source: Bioinformatics. 2021 Jul 12;37(Suppl 1):i289–98. doi: 10.1093/bioinformatics/btab288 (PMC8336595; doi:10.1093/bioinformatics/btab288)
Supplement: btab288_Supplementary_Data [file btab288_supplementary_data.pdf]

Supplementary Materials
JEDI: Circular RNA Prediction based on Junction Encoders and Deep Interaction among Splice Sites

Jyun-Yu Jiang[1], Chelsea J.-T. Ju[1], Junheng Hao[1], Muhao Chen[2], and Wei Wang[1]

[1]Department of Computer Science, University of California, Los Angeles, CA 90095, USA
[2]Department of Computer Science, University of Southern California, Los Angeles, CA 90007, USA

S1 Analysis on the Cross-attention Layer

The cross-attention layer plays an essential role in our proposed method to derive cross-attentive acceptor and donor embeddings. To further study how the cross-attention layer benefits the task of circular RNA prediction, given the embedding of each donor as a query, we conduct an analysis to rank all acceptors by their embedding similarity to the query embedding. Conversely, we also rank all donors for each acceptor. This analysis can be considered as a ranking task, so we evaluate the results with three conventional ranking metrics, including success rate at 10 (SR@10), mean average precision (MAP), and mean reciprocal rank (MRR), based on the ground-truth of backsplicing for gene-level circular RNA prediction. Here we compare our results with a random ranker as a chance-level baseline. As shown in Table S1, JEDI can significantly beat the chance performance in both acceptor and donor ranking tasks. Besides, it is interesting that donor embeddings outperform acceptor embeddings in retrieving the opposite sites. It may be because donor embeddings contain more information than acceptor embeddings. We remain the study of this phenomenon as future work.

Query Embedding	Ranking Candidates	JEDI			Random (Chance-level Performance)		
		SR@10	MAP	MRR	SR@10	MAP	MRR
Donor	Acceptor	0.843 ± 0.013	0.375 ± 0.013	0.394 ± 0.012	0.432 ± 0.004	0.128 ± 0.002	0.171 ± 0.003
Acceptor	Donor	0.571 ± 0.048	0.189 ± 0.026	0.202 ± 0.032	0.432 ± 0.006	0.123 ± 0.002	0.172 ± 0.003

Table S1. The results of ranking acceptors and donors with opposite embeddings.

S2 Analysis on *k*-mer Embedding Dimension

Figure S1 shows how the k-mer embedding dimension affects the prediction performance. JEDI reaches the best performance when it comes to 128-dimensional k-mer embeddings. However, the prediction accuracy drops with greater embedding dimension numbers because of the over-fitting phenomenon with excessive model parameters. Finally, we choose 128 as the dimension number for k-mer embeddings.

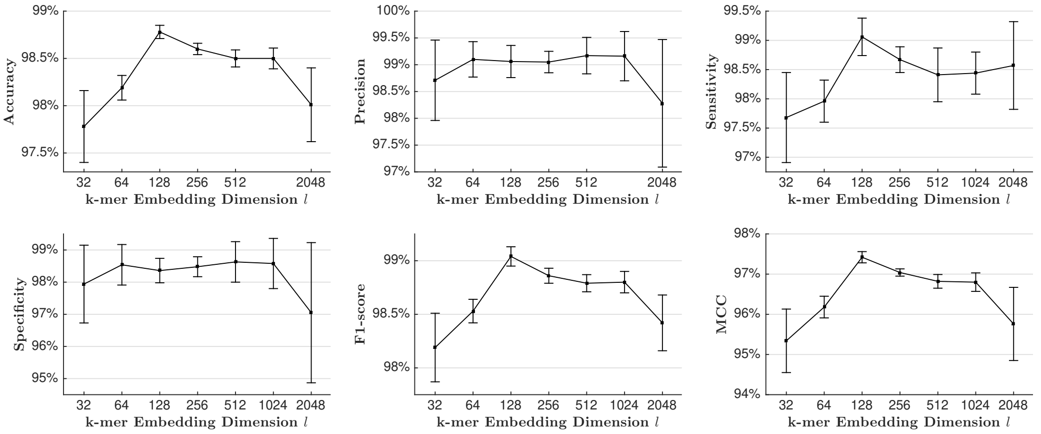

Fig. S1. The isoform-level circular RNA prediction performance of JEDI with different k-mer embedding dimension number l.
